# IgM N-glycosylation correlates with COVID-19 severity and rate of complement deposition

**DOI:** 10.21203/rs.3.rs-2939468/v1

**Published:** 2023-06-02

**Authors:** Benjamin Haslund-Gourley, Kyra Woloszcuk, Jintong Hou, Jennifer Connors, Gina Cusimano, Mathew Bell, Bhavani Taramangalam, Slim Fourati, Nathan Mege, Mariana Bernui, Matthew Altman, Florian Krammer, Harm van Bakel, Holden Maecker, Brian Wigdahl, Charles Cairns, Elias Haddad, Mary Comunale

**Affiliations:** Drexel University College of Medicine; Drexel University College of Medicine; Drexel University College of Medicine; Drexel University College of Medicine; Drexel University College of Medicine; Drexel University College of Medicine; Drexel University College of Medicine; Drexel University College of Medicine; Drexel University College of Medicine; Drexel University College of Medicine; Benaroya Research Institute; Icahn School of Medicine at Mount Sinai; Icahn School of Medicine at Mount Sinai; Stanford University School of Medicine; Drexel University College of Medicine; Drexel University/Tower Health Hospital; Drexel University; Drexel University College of Medicine

**Keywords:** COVID-19, SARS-CoV-2, IgM N-glycan, Immunoglobulin M, Glycomics, Complement Deposition

## Abstract

The glycosylation of IgG plays a critical role during human SARS-CoV-2, activating immune cells and inducing cytokine production. However, the role of IgM N-glycosylation has not been studied during acute viral infection in humans. *In vitro* evidence suggests that the glycosylation of IgM inhibits T cell proliferation and alters complement activation rates. The analysis of IgM N-glycosylation from healthy controls and hospitalized COVID-19 patients reveals that mannosylation and sialyation levels associate with COVID-19 severity. Specifically, we find increased di- and tri-sialylated glycans and altered mannose glycans in total serum IgM in severe COVID-19 patients when compared to moderate COVID-19 patients. This is in direct contrast with the decrease of sialic acid found on the serum IgG from the same cohorts. Moreover, the degree of mannosylation and sialylation correlated significantly with markers of disease severity: D-dimer, BUN, creatinine, potassium, and early anti-COVID-19 amounts of IgG, IgA, and IgM. Further, IL-16 and IL-18 cytokines showed similar trends with the amount of mannose and sialic acid present on IgM, implicating these cytokines’ potential to impact glycosyltransferase expression during IgM production. When examining PBMC mRNA transcripts, we observe a decrease in the expression of Golgi mannosidases that correlates with the overall reduction in mannose processing we detect in the IgM N-glycosylation profile. Importantly, we found that IgM contains alpha-2,3 linked sialic acids in addition to the previously reported alpha-2,6 linkage. We also report that antigen-specific IgM antibody-dependent complement deposition is elevated in severe COVID-19 patients. Taken together, this work links the immunoglobulin M N-glycosylation with COVID-19 severity and highlights the need to understand the connection between IgM glycosylation and downstream immune function during human disease.

## 1. Introduction

SARS-CoV-2 (COVID-19) has impacted the world significantly since its outbreak in late 2019, killing more than 14 million between 2020–21 [1]. Once viral particles are inhaled and enter the human airway, the spike (S) protein trimer expressed on the surface of SARS-CoV-2 membranes binds and infects cells via the angiotensin-converting enzyme 2 (ACE2) abundant in airway epithelial and endothelial cells [2]. The resulting infection consists of two overlapping phases. The first mainly consists of viral replication associated with mild constitutional symptoms. During the second phase, a combination of the host’s adaptive and innate immune response can result in either the efficient clearance of virus-infected cells or the induction of multi-organ system damage requiring intensive care [3]. Patients in this second phase with severe COVID-19 often present with elevated D-dimer [4], C-reactive protein (CRP) [5], IL-6 [6], acute kidney injury [7], and heightened complement deposition [8, 9].

Immunophenotyping assessment in a COVID-19 cohort (IMPACC) was designed at the beginning of the pandemic with the intent to enroll hospitalized patients with COVID-19 to collect detailed clinical, laboratory and radiography data with the intent of turning this into a prospective longitudinal study [10]. Biological samples including blood, nasal swabs, and endotracheal aspirates were collected at multiple time points during hospitalization. Five trajectory time points were identified previously based on clinical data from the entire IMPACC cohort. Patient trajectories were divided into 5 groups based on longitudinal observation of ordinal scores reflecting the degrees of respiratory illness and presence or absence of complications at discharge [11]. Trajectory Group 1 was characterized by a brief hospital stay of 3–5 days without major complications. Trajectory 2 had a longer length of stay (7–14 days) with no complications upon discharge. Trajectory 3 was characterized by an intermediate length of stay (10–14 days) with limitations at discharge. The most severe trajectory groups are 4 and 5. Trajectory 4 had a longer length of stay (~ 28) days with complications, while Trajectory 5 was characterized by fatal illness by day 28. Thus, the curation and stratification of these samples provided an opportunity to determine how human glycosylation relates to acute COVID-19 infection severity.

The glycosylation of immunoglobulins plays an important role during the adaptive immune response to infection and vaccination [12–15]. IgG is the best example of how variations in immunoglobulin glycosylation modulate downstream immune responses. The size and charge of IgG N-glycans occupying Asn-297 site of the Fc heavy chain can promote antibody-dependent cellular-cytotoxicity (ADCC), antibody-dependent cellular phagocytosis (ADCP), Fc-gamma receptor affinity [16–21], and complement activation [20, 22, 23]. In hospitalized COVID-19 patients, the sialic acid and galactose content on total IgG N-glycans was reduced compared to patients with mild cases of COVID-19 and healthy controls [17–19]. Furthermore, anti-spike IgG isolated from hospitalized COVID-19 patients contained lowered core-fucose levels in severe patients [24–28], promoting macrophage release of IL-6 and TNF-α and the destruction of endothelial barriers *in vitro* by binding FcγR IIA and IIIA [29].

While much attention has been paid to the glycosylation of IgG, less has been focused on IgM. IgM is the third most abundant circulating immunoglobulin and is produced early during the adaptive immune response to COVID-19 infection [30]. Moreover, IgM is a highly potent immune protein. A single immune-complexed IgM can initiate the complement cascade [31] and plays important roles during early immune responses to clear bacteria, viruses, parasites, apoptotic cells, and are likely involved in promoting immune tolerance [32]. The heavy chain of IgM contains five separate N-glycosylation sites containing both complex-type, hybrid N-glycans, and highly-mannosylated N-glycans [33, 34]. Complex type N-glycans populate IgM at Asn-171, Asn-332, and Asn-395 while Asn-402 and Asn-563, located closer to the tail of the IgM heavy chain, are populated with mannose N-glycans [34, 35]. In cell lines treated with tunicamycin to block glycosylation of IgM, secretion of IgM fell by > 95% [36], demonstrating N-glycan’s crucial role in the secretion of IgM from B-cells. The distinct regions of complex-type and mannose-rich N-glycosylation on the heavy chain of IgM have been reported to occupy different ‘faces’ or sides of IgM with the complex-type N-glycans participating in binding antigens. *In vivo*, increased IgM sialylation was associated with heightened T cell inhibition [37]. In addition, evidence supports IgM N-glycans participating in both the classical and lectin complement pathways [38, 39]. The recently discovered IgM-specific receptor, FcμR, expressed on NK, B, and T cells has implicated IgM in controlling cellular activation and antibody production [40]. Additional receptors for IgM Fc include Fcα/μR expressed by germinal center follicular dendritic cells [41] and pIgR requiring the J-chain pentamer of IgM for transcytosis into mucosal surfaces [42]. However, the function of IgM N-glycans interacting with these receptors remains to be explored.

While the N-glycosylation of IgM has been characterized previously in healthy pooled human serum, during cancer [35, 43–45], and in recombinant IgM [34, 46], this is the first characterization of the IgM N-glycosylation profile isolated from humans infected with an acute viral disease. Here, we report significant differences in the IgM N-glycan content from cohorts of hospitalized COVID-19 separated by severity trajectory. Total mannosylation decreased while di-sialylation (S2) increased on IgM – opposing the trend detected in the same cohorts of reduced IgG sialylation. Moreover, glycosylation of IgM correlates with circulating immune cell glycosyltransferase expression of *ST3GAL4* and *MAN1A2*, previously reported clinical markers of COVID-19 severity, and elevations in cytokines IL-16 and IL-18. Lastly, we report an increased antibody-dependent complement deposition induced by IgM from the severe COVID-19 cohort.

## 2. Methods

### Human samples

#### Patient enrollment and consent

The IMPACC is a collaborative project developed by the NIAID and investigators from the Human Immunology Project Consortium (HIPC), the Asthma and Allergic Diseases, and the Cooperative Disease Research Centers (AADCRC). Drexel University collected 106 patient samples to be included in the IMPACC through the Tower Health Hospital network. Participants are enrolled within 48 hours of hospitalization where demographics, detailed medical history, and clinical data were taken. Consenting participants are enrolled within 48 hours of hospitalization under the IRB Protocols 2004007753 and 2102008337. Upon enrollment, demographics, COVID-19 symptom onset, detailed medical history (including comorbidities), and medical records were all recorded. Patients were confirmed positive with a SARS-CoV-2 polymerase chain reaction (PCR). Extensive clinical labs are taken during intake- and biological samples including blood, nasal swab, and endotracheal aspirates are collected. Clinical data and samples from days 4 and 7, representing patient admission to the hospital, were examined.

#### Biological Sample Processing

Blood samples and nasal swabs were collected at each timepoint and processed at Drexel University within 6 hours of collection according to the IMPACC standardized operating procedure [10]. Whole blood, nasal swabs, peripheral blood mononuclear cells (PMBCs), and plasma collected from each patient was processed at Drexel University and sent to IMPACC core facility sites for further analysis as previously reported [10, 11]. PBMCs were used to identify immune cell populations and changes in cell populations, gene expression, and activation markers. Plasma was used to characterize antibody titers, anti-RBD titers, antibody isotype, proteomics, and metabolomics. At Drexel, plasma was additionally used for ELISA antibody abundance analysis, Luminex cytokine and chemokine assays, and glycomic analysis. Whole blood was used in genome-wide association study (GWAS) and cytometry by the time-of-flight (CyTOF) and bulk RNA transcriptomics. Nasal Swabs were used for bulk RNAseq and viral load quantitaion.

##### PBMC Isolation:

Patient blood samples were spun down at 1000 × g for 10 minutes at room temperature, and plasma was aliquoted. The remaining blood was diluted 1:2 with DPBS (Ca^+ 2^Mg^+ 2^ free) and slowly pipetted into a 50mL SepMate-50 tube (with 15mL Lymphoprep below the insert). Samples were spun at 800 × g for 20 minutes at 20°C with brakes off. The top layer with PBMCs was transferred to a new tube and cells were washed at 400 × g for 5 minutes. Cells were resuspended in 20mL EasySep Buffer, then spun again at 300 × g for 10 minutes at room temperature. For RNASeq, cells were resuspended at 5 million per mL, and 50uL was aliquoted into CRYSTAL Gen tubes. Cells were spun at 500 × g for 5 minutes at room temperature and the excess media was removed. 200uL QIAGEN RLT Buffer with (BME) was added and vortex until the pellet was fully dissolved. Samples were stored at −80°C for shipment. The remaining PBMCs were frozen down in FBS + DMSO for storage at Drexel University.

#### Anti-SARS-CoV-2 nucleocapsid IgA, IgG, and IgM quantitation

Monobind AccuBind^®^ ELISA Anti-SARS-CoV-2 kits were used as a qualitative determination of Anti-SARS-CoV-2 specific IgA, IgG and IgM antibodies at Drexel’s IMPACC site. These kits utilize a sequential sandwich ELISA method. This test utilizes recombinant nucleocapsid protein (rNCP) from SARS-CoV-2 coated on microwells to capture antibodies in human plasma. Patient plasma was diluted 1:100 and added directly to the ELISA plate. Following incubation and washing, IgA, IgG or IgM labeled antibodies were added. After a second incubation and wash, reagent substrate is added to produce a measurable color through the reaction with enzyme and hydrogen peroxide. After the addition of a stop substrate, absorbance was read in each well at 450nm within 15 minutes of adding the stop solution.

#### Cytokine and chemokine analysis

Patient plasma was analyzed for chemokine/cytokine levels using the human immune monitoring 65-Plex ProcartaPlex^™^ Panel (Invitrogen^™^). This kit was used to determine the levels of 65 cytokines, chemokines, growth factors, and soluble receptors produced at the designated time points at the Drexel IMPACC site. The following human chemokine/cytokine premixed panel was used according to the manufacturer’s protocol: G-CSF (CSF-3), GM-CSF, IFN alpha, IFN-g, IL-1a, IL-1b, IL-2, IL-3, IL-4, IL-5, IL-6, IL-7, IL-8 (CXCL8), IL-9, IL-10, IL-12p70, IL-13, IL-15, IL-16, IL-17A (CTLA-8), IL-18, IL-20, IL-21, IL-22, IL-23, IL-27, IL-31, LIF, M-CSF, MIF, TNF-a, TNF-b, TSLP, BLC (CXCL13), ENA-78 (CXCL5), Eotaxin (CCL11), Eotaxin-2 (CCL24), Eotaxin-3 (CCL26), Fractalkine (CX3CL1), Gro-alpha (CXCL1), IP-10 (CXCL10), I-TAC (CXCL11), MCP-1 (CCL2), MCP-2 (CCL8), MCP-3 (CCL7), MDC (CCL22), MIG (CXCL9), MIP-1a (CCL3), MIP-1b (CCL4), MIP-3a (CCL20), SDF-1a (CXCL12), FGF-2, HGF, MMP-1, NGF-b, SCF, VEGF-A, APRIL, BAFF, CD30, CD40L (CD154), IL-2R (CD25), TNF-RII, TRAIL (CD253), TWEAK. Data was acquired on a Luminex^™^ FLEXMAP 3D^™^ System using bead regions defined in the protocol and analyzed using Belysa Curve Fitting Software (Sigma Aldrich). Standard curves were generated, and sample concentrations were calculated in pg/mL.

### Nasal viral PCR, host transcriptomics, and metagenomics

#### RNA preparation

Inferior nasal turbinate swabs were collected and placed in 1ml of Zymo-DNA/RNA shield reagent (Zymo Research). RNA was extracted from 250 μL of sample and eluted into a volume of 50ul using the KingFisher Flex sample purification system (ThermoFisher) and the quick DNA-RNA MagBead kit (Zymo Research) following the manufacturer’s instructions. Each sample was extracted twice in parallel. The 2 eluted RNA samples were pooled and aliquoted into 20 μL aliquots using a Rainin Liquidator 96 pipettor for downstream RT-qPCR, RNA-sequencing, and viral sequencing.

##### RealTime Quantitative Polymerase Chain Reaction:

Master mixes containing nuclease-free water, combined primer/probe mixes, and One-Step RT675 qPCR ToughMix (Quantabio) were prepared on ice, and 15 μL was dispensed in each well of a 384-reaction plate (Thermofisher) CoV2 was quantitated using the CDC qRT-PCR assay (primers and probes from IDT). Briefly, this comprises two reactions targeting the CoV2 nucleocapsid gene (N1 and N2) and one reaction targeting RPP30 (RP). Each batch included positive controls of plasmids containing N1/N2 and RP target sequence (2019-nCoV_N_Positive Control and Hs_RPP30 Positive Control, IDT) to allow quantitation of each transcript. Primer/probe sequences were: 2019-nCOV_N1-F GAC CCC AAA ATC AGC GAA AT, 2019-nCOV_N1-R TCT GGT TAC TGC CAG TTG AAT CTG, 2019-nCOV_N1-P ACC CCG CAT TAC GTT TGG TGG ACC, 2019-nCOV_N2-F TTA CAA ACA TTG GCC GCA AA, 2019-nCOV_N2-R GCG CGA CAT TCC GAA GAA, 2019-nCOV_N2-P ACA ATT TGC CCC CAG CGC TTC AG, RP-F AGA TTT GGA CCT GCG AGC G, RP-R GAG CGG CTG TCT CCA CAA GT and RP-P TTC TGA CCT GAA GGC TCT GCG CG. After RNA extracts were gently vortexed and added 5 μL per sample. Plates were centrifuged for 30 seconds at 500 × g, 4C. The quantitative polymerase chain reaction was performed using a Quantstudio5 (Thermo Fisher) with cycling conditions: 1 cycle 10 min at 50°C, followed by 689 3 min at 95°C, 45 cycles 3 sec at 95°C, followed by 30 sec at 55.0°C.

##### RNA-sequencing cDNA Library Production:

From each nasal RNA sample, 10ul was aliquoted to a library construction plate using the Perkin 692 Elmer Janus Workstation (Perkin Elmer, Janus II). Ribosomal depletion, cDNA synthesis, and library construction steps were performed using the Total Stranded RNA Prep with Ribo-Zero Plus kit, following the manufacturer’s instructions (Illumina). All steps were automated on the Perkin Elmer Sciclone NGSx Workstation to reduce batch-to-batch variability and increase sample throughput. Final cDNA libraries were quantified using the Quant-it dsDNA High Sensitivity assay, and library insert size distribution was checked using a fragment analyzer (Advanced Analytical; kit ID DNF474). Samples, where adapter dimers constituted more than 4% of the electropherogram area, were failed before sequencing. Technical controls (K562, Thermo Fisher Scientific, cat# AM7832) were compared to expected results to ensure that batch-to-batch variability was minimized. Successful libraries were normalized to 10nM for sequencing.

### RNA-sequencing Clustering and Sequencing

Barcoded libraries were pooled using liquid handling robotics prior to loading. Massively parallel sequencing-by-synthesis with fluorescently labeled, reversibly terminating nucleotides was carried out on the NovaSeq 6000 sequencer using S4 flowcells with a target depth of 50 million 100 base-pair paired-end reads per sample (25 million read pairs).

#### Total IgG isolation

Total IgG was isolated from 20μL of plasma using a Protein G spin plate as described by the manufacturer (ThermoFisher, MA). Four 200μL 1X PBS washes removed unbound plasma protein using a vacuum manifold apparatus. Next, IgG was eluted by incubating 150μL of 0.1M glycine HCl pH 2–3 for 5 minutes at room temperature. The eluate was collected into a 96-well 2mL collection plate pre-loaded with 15μL of 1.5M Tris pH 8 to neutralize the glycine elution buffer. The wash process was repeated a second time to ensure a high yield of IgG. The resulting 315μL of the neutralized eluate was concentrated and buffer-exchanged to 20μL of 1X PBS using Amicron Ultra-0.5 centrifugal Filter 10 kDa MWCO (Millipore) following the manufacturer’s instructions. NanoDrop 1000 spectrophotometer readings monitored protein yield through the isolation process.

#### Total IgM isolation

Total IgM was isolated from plasma by incubating 80μL of goat anti-IgM agarose-conjugated agarose beads (A9935, Millipore Sigma, MA) with 80μL plasma and 100μL 1X PBS for 2 hours at room temperature. Following the incubation, the solution was transferred to a 1.2um MultiScreen HTS 96-well filter plate. Four 200μL 1X PBS washes removed unbound plasma protein using a vacuum manifold apparatus. Next, IgM was eluted by incubating 150μL of 0.1M glycine HCl pH 2–3 for 5 minutes at room temperature. The eluate was collected into a 96-wel 2mL collection plate pre-loaded with 15μL of 1.5M Tris pH 8 to neutralize the glycine elution buffer. The wash process was repeated a second time to ensure a high yield of IgM. The resulting 315μL of the neutralized eluate was concentrated and buffer-exchanged to 20μL of 1X PBS using Amicron Ultra-0.5 centrifugal Filter 10 kDa MWCO (Millipore) following the manufacturer’s instructions. NanoDrop 1000 spectrophotometer readings monitored protein yield through the isolation process

#### Immunoglobulin N-glycan analysis

N-glycans from IgG and IgM were released, labeled, and analyzed as described previously using the Waters GlycoWorks RapiFluor MS kit, adapted for PCR tubes [47]. Briefly, samples were denatured using the RapiGest reagent for 5 minutes at 95°C using a PCR thermocycler. Next, glycoprotein samples were deglycosylated using PNGase F for 6 minutes at 60°C using a PCR thermocycler. Afterward, samples were labeled with RapiFluor label (RFMS) for 5 minutes at room temperature. A solid-phase extraction (SPE) clean-up module isolated RFMS labeled N-glycans which were then eluted into a 96-well 2mL Waters ANSI plate capped with a PFTE 96-well membrane top for high-throughput N-glycan analysis. An ACQUITY Premier UPLC System was used following the setting and protocol described previously [47]. Briefly, a ACQUITY UPLC BEH Amide Column, 130Å, 1.7 μm, 2.1 mm X 50 mm column (Waters, MA) was used to chromatographically separate N-glycans during the 18.3 min run employing a gradient of 50mM Ammonium Formate pH 4.4 (Waters) made with LC-MS Water (Millipore), LC-MS ACN (VWR, Honeywell) 25%−75% gradient transitioning over 12 min to 60%−40%. N-glycans separated by charge and stereochemistry were quantitated using Waters AQUITY Fluorescent detector set to 265/425 em/ex, 10Hz using Empower 3 software. Lastly, N-glycan identity was confirmed using a Waters AQUITY QDa Mass spectrometer. The resulting UPLC fluorescent trace was analyzed with Empower v3.3.1 software, UPLC trace percent-area was combined with collected MS-spectra to identify eluted peaks as described previously [47]. Pooled N-glycans labeled with the RapiFluor tag were digested with Neuraminidase S (New England BioLabs, MA, P0743L) or Neuraminidase (New England BioLabs, MA, P0720S) for 12 hours at 32°C following the manufacturer’s instructions. Digested N-glycans were cleaned up using Water’s SPE kit and analyzed using the UPLC detailed above.

### Antigen-specific complement deposition assay

Antibody-specific complement deposition against the RBD and Spike S1 antigens were assayed following the previously developed protocol [48]. Briefly, 20μL FluoSpheres^™^ NeutrAvidin^™^-Labeled Microspheres (ThermoFisher) were incubated with 20μg RBD (aa319–541, Invitrogen) (biotinylated in-house using the EZ-Link^™^ Sulfo-NHS-LC-Biotinylation Kit) or 20μg biotinylated SARS-CoV-2 (2019-nCoV) Spike S1-His Recombinant Protein, Biotinylated (SinoBiological) antigen for 4 hours at 37°C. After washing twice with 200μL 1X PBS, the antigen-bound beads were blocked with 200μL 5% BSA in 1X PBS for 1 hour at 37°C. Next, the beads were washed twice with 500μL of 0.1% BSA in 1X PBS and diluted 1:100 in 1X PBS. A subset of plasma and purified IgM samples were treated with either a Mannosidase (New England BioLabs, MA, P0768S) or Neuraminidase (New England BioLabs, MA, P0720S) for 12 hours at 32°C prior to antigen-specific complement deposition analysis following the manufacturer’s instructions. Next, 15μL of the 1:100 bead solution was transferred to low-binding 1.5mL tubes (Corning) and incubated with 20μL of 1:10 1X PBS diluted pooled severe or nonsevere plasma or 5μg of IgM isolated from pooled severe or nonsevere plasma for 2 hours at 37°C. Next, the immune-complexed beads were incubated for 15 minutes with freshly resuspended Guinea pig complement (Cedarlane, CL4051) and diluted 1:50 in Gelatin Veronal Buffer with Mg2+ & Ca2+ (GVB++) at 37°C. The complement deposition was halted with two washes of 200μL 15mM EDTA. Next, 50μL of a 1:100 diluted FITC labeled Goat anti-Guinea pig Complement C3 antibody (MP Biomedicals, 085538) was incubated for 30 minutes with the immune-complexed beads. Lastly, two 200μL 1X PBS washes removed unbound FITC labeled anti-C3 antibody. Washed samples were re-suspended in 100uL and analyzed using a Fortessa Flow Cytometer (BD). Beads were gated for the presence or absence of the FITC antibody, and the MFI of the bead content was divided by the total number of beads to determine the rate of complement deposition in each sample. The gating strategy is displayed in Fig. 4B. Flow Minus One (FMO) control samples were run with the same protocol to confirm a low background signal and inform the gating cut-off strategy.

### Statistical analysis

A biomarker was removed from analysis if its overall number of missing values was greater than 3 (13.6% of 22 patients) to reduce potential bias [49–51]. Data analysis was performed using R and GraphPad Prism 8. COVID-19 trajectory groups were categorized as “1–3” and “4–5” for the averages of measured transcriptomic, proteomic, Luminex, and clinical data. Gender and COVID-19 trajectory group categories were summarized as counts and percentages, continuous variables were summarized as the median and interquartile range (IQR) overall and by trajectory group category. For transcriptomic data, raw counts were normalized to counts per million (CPM), then values were log2 transformed for statistical analysis. A pseudo-count of 2 was added to all count data prior to log transformation because zero cannot be ‘logged’ [52–54]. Mann-Whitney U test was used to test the significance of continuous variables between trajectory group categories. A chi-square test was used to test the association between gender and trajectory group category. Associations between IgM Mannosylated or total S2 and other variables were tested using simple linear regression. Raw trajectory group values were used in simple linear regression. Coefficient of determination R2 was obtained from linear regression. p < 0.05 was considered statistically significant for all tests.

## 3. Results

### IgM di-sialylation and mannosylation associate with COVID-19 severity

**Figure 1:**
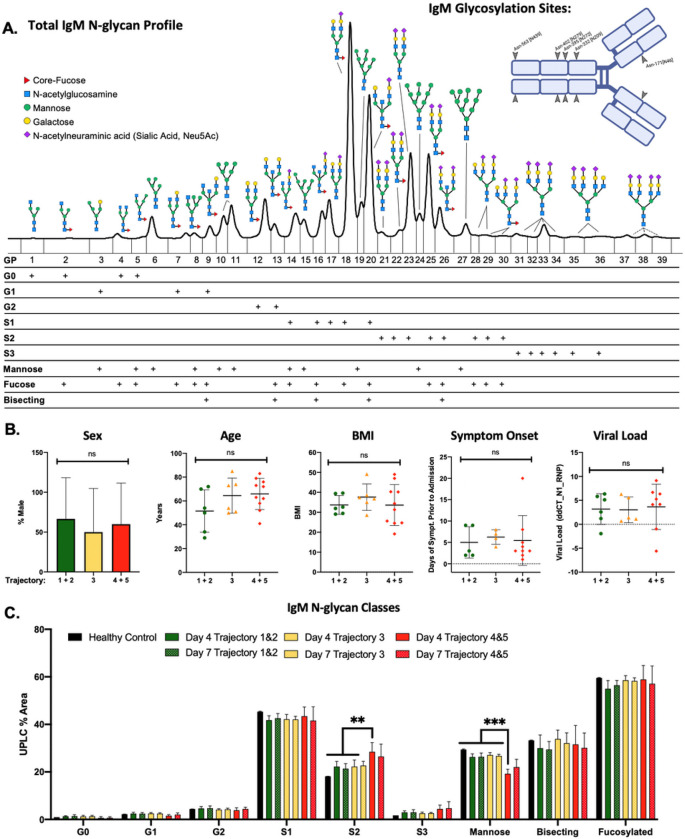
IgM N-glycosylation analysis reveals differences in COVID-19 patients stratified by trajectory **A)** IgM N-glycans labeled with the RapiFluor (RFMS) were profiled with UPLC-FLR-ESI-MS. The resulting N-glycans were identified using mass spectrometry and retention time data. Please see Supplementary Table 1 for a complete list of N-glycans. Dashed lines represent N-glycans without confirmed mass identities due to the limitation of the RFMS label in the QDa mass spectrometer. IgM monomer is displayed with the 5 conserved glycosylation sites labeled **B)** Cohort demographics: Sex, age, body mass index (BMI), time from symptom onset to hospital admission, and viral load expressed as the delta-delta change between SARS-CoV-2 Nucleocapsid protein 1 (N1) and the house keeping gene RNP via RT qPCR are presented stratified across trajectory 1–2, 3, and 4–5 C) IgM N-glycans are grouped by class: G0 refers to core diantennary N-glycans lacking galactose, G1 refers to core diantennary N-glycans with a single galactose, G2 refers to core diantennary N-glycans with two galactoses, S1 refers to diantennary N-glycans with a single sialic acid, S2 refers to di- and tri-antennary N-glycans with two sialic acids, S3 refers to triantennary N-glycans with three sialic acids, Mannose refers to M4-M10 and hybrid-type N-glycans, Bisecting refers to any N-glycan with a bisecting GlcNAc moiety, Fucosylated refers to any N-glycan with a core-fucose. Healthy Control (n=2), Day 4 Trajectory 1&2 (n=6), Day 7 Trajectory 1&2 (n=5), Day 4 Trajectory 3 (n=6), Day 7 Trajectory 3 (n=5), Day 4 Trajectory 4&5 (n=10), Day 7 Trajectory 4&5 (n=5). N-glycan classes listed in the above graph +/− S.D. with significance denoted were analyzed using a two-way ANOVA with Tukey’s multiple comparisons test **p < 0.01, ***p < 0.001.

Plasma from patients admitted to the hospital after testing positive for COVID-19 was analyzed 4- and 7-days post-admission. Clinical characteristics of the patients are presented in Table 1 stratified by trajectory 1–5, with 1 being a mild COVID-19 infection and 5 being death from complications of COVID-19 infection. N-glycan profiles isolated from purified total IgM were analyzed (Fig. 1A), with N-glycan identities listed in **Supplemental Table 1**. N-glycans ranging from mono-antennary to tri-antennary as well as hybrid and mannosylated moieties were observed in all IgM samples. The 36 individual IgM N-glycan peaks with identities confirmed by mass-spectrometry from day 4 and day 7 are included in **Supplemental Figs. 1 and 2**. To analyze general trends in the IgM N-glycan profile across disease severity, glycans were grouped by size, charge, and type into classes (G0, G1, G2, S1, ect.) as denoted below the IgM N-glycan profile in Fig. 1A.

Protein glycosylation is impacted by factors including sex, age, and BMI [55–66]. Therefore, COVID-19 patient cohorts from the IMPACC study were analyzed to determine if there were statistically significant differences between mild (trajectories 1 and 2), moderate (trajectory 3), and severe (trajectories 4 and 5) (Fig. 1B). There was no statistically significant difference between cohorts based on sex, age, BMI, the number of days of COVID-19 symptoms prior to hospitalization, or viral load. Furthermore, we determined there was no statistically significant difference in the concentration of total IgM isolated between each patient cohort (**Supplemental Fig. 3**). After confirming that cohort characteristics were comparable, we analyzed the IgM N-glycosylation profiles from day 4 and 7 hospitalized COVID-19 IMPACC patients across mild, moderate, and severe cohorts (Fig. 1C). Di-sialylated (S2) N-glycans on IgM increased significantly in the severe COVID-19 cohort on day 4 of hospitalization compared to the mild and moderate cohorts. In addition, total mannose, including hybrid N-glycans, decreased significantly in the severe COVID-19 cohort on day 4 IgM. On day 7, the severe cohort’s IgM N-glycosylation maintained the trends observed on day 4, but lost significance likely due to the death of four of the COVID-19 patients in the severe trajectories reducing the power of the analysis. Taken together, the changes in IgM N-glycosylation correlate with the severity of COVID-19 infection in humans.

### IgG and IgM N-glycans responses differ during COVID-19

**Figure 2:**
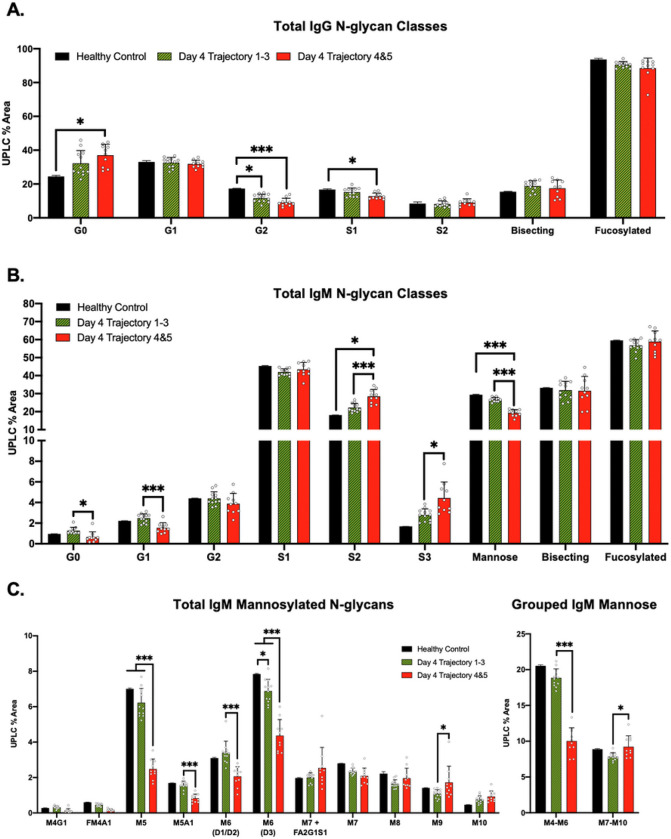
IgM N-glycan profile stratifies cohorts of nonsevere from severe trajectory COVID-19 patients **A)** IgG N-glycans from healthy control (n=2), day 4 trajectory 1–3 (n=12), and day 4 trajectory 4&5 (n=10) cohorts. N-glycans are graphed as grouped classes – see supplemental figure 4 for a full list of N-glycans and N-glycan grouping. **B)** IgM N-glycan profiles from cohorts of healthy control (n=2), day 4 trajectory 1–3 (n=12), and day 4 trajectory 4&5 (n=10) hospitalized COVID patients. See Figure 2A for a detailed explanation of the N-glycan classes. **C)**IgM mannosylated N-glycans from non-severe compared to severe COVID-19. A summation of the indicated mannose/hybrid N-glycan sub-groups are graphed to the right. IgM N-glycan classes graphed as mean +/− S.D. with significance determined using multiple unpaired T-tests *p < 0.05, **p < 0.01, ***p < 0.001

We next compared the glycosylation of bulk IgM and IgG isolated from COVID-19 patients to characterize the plasma blast glycosylation response to viral infection. Patients were sorted into nonsevere (trajectories 1–3) and severe (trajectories 4 and 5) cohorts to compare the change in immunoglobulin N-glycosylation by glycan class. First, IgG N-glycans from healthy control, nonsevere, and severe COVID-19 cohorts were analyzed as grouped classes (G0, G1, G2 ect.) as described in **Supplemental Fig. 4**. IgG in both severe and nonsevere COVID-19 exhibited reduced di-galactosylation (G2) and mono-sialylation (S1) while agalactosylation (G0) significantly increased compared to healthy controls in the severe COVID-19 cohort (Fig. 2A). Interestingly, the IgG N-glycosylation of the severe and nonsevere cohorts did not exhibit statistically significant differences between one another. In contrast, the IgM glycosylation from the same patients revealed statistically significant changes between severe and nonsevere cohorts (Fig. 2B). Agalactosylated (G0) and mono-galactosylated (G1) N-glycans significantly decreased in severe patients compared to the nonsevere cohort. Further, the increase in S2 remained significant while tri-sialylated (S3) content also increased significantly in the severe COVID-19 cohort. In comparison, the sialyation of severe patient IgG N-glycans remained lowered or unchanged on day 4 compared to healthy controls (Fig. 2A), aligning with previous studies of IgG N-glycosylation in hospitalized COVID-19 patients [24, 27, 28]. Lastly, the decrease in mannose remained significant in severe trajectory patients compared to nonsevere patients on day 4 of hospitalization.

The decrease in total mannose content required further interrogation because 11 hybrid and mannosylated N-glycans contribute to the overall decrease observed in the IgM during severe COVID-19 (Fig. 2C). The decrease in total mannose was predominantly due to lowered levels of the smaller hybrid moieties: M4G1, FM4A1, and M5A1 in combination with the mannosylated moieties: M5 and the two isoforms of M6. Mannosylated structures or co-eluting peaks larger than M6 did not significantly decrease, while M9 significantly increased in the severe COVID-19 cohort. Next, mannose and hybrid structures ranging from M4-M6 were compared to mannose structures M7-M10, revealing a potential reduction in the degree of mannose processing by Golgi-bound mannosidases during IgM production. Taken together, the glycosylation pattern of IgM was consistently altered in the severe COVID-19 cohort, with major classes of IgM N-glycans trending in opposite directions compared to the IgG N-glycan classes.

### Glycosyltransferase expression correlates with IgM N-glycosylation

The observed changes in IgM N-glycosylation likely result from glycosyltransferase expression within the Golgi of plasmablasts. The IMPACC study collaborators at Emory University provided 61 glycosyltransferase and glycosidase transcript expression data isolated from peripheral blood mononuclear cells (PBMCs) collected on day 0 of patient hospitalization. After normalizing the data by total read count and transforming by log2 for comparability, expression profiles were compared between the severe and nonsevere COVID-19 cohorts.

**Figure 3:**
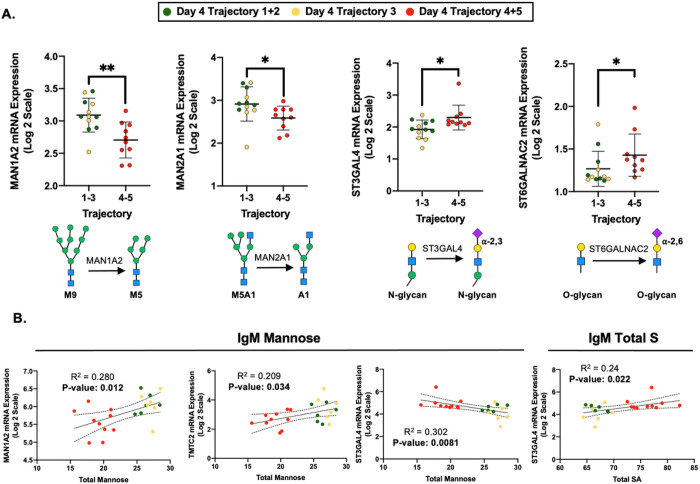
Changes in IgM N-glycosylation correlate with PBMC glycosyltransferase/glycosidase mRNA expression **A)** Expression of MAN1A2, MAN2A1, ST3GAL4, and ST6GALNAC2 were significantly different between the COVID-19 trajectory 1–3 (nonsevere) and trajectory 4 and 5 (severe). The role of each glycosidase and glycosyltransferase are depicted below. **B)** Total mannose on IgM positively correlated with MAN1A2 and TMTC3 expression while negatively correlating with ST3GAL4 expression. The summation of sialic acids on IgM positively correlated with ST3GAL4 expression.

The expression of the mannosidases MAN1A2 and MAN2A1 decreased significantly in the severe cohort compared to the nonsevere cohort (Fig. 3A). These mannosidases are responsible for processing high mannose structures into smaller mannose moieties [67]. The decrease in mannosidase expression aligns with data in Fig. 2C where we observe less mannosidase-processed M5 and M6 content in the severe COVID-19 cohort IgM. In addition, IgM total mannose correlated with MAN1A2, the o-mannosyltransferase TMTC2, and the α−2,3 sialyltransferase ST3GAL4 (Fig. 3B).

The expression of the α−2,3 sialyltransferase ST3GAL4 and the O-glycan α−2,6 sialyltransferase ST6GALNAC2 were significantly elevated in the severe COVID-19 cohort (Fig. 3A). Interestingly, the ST6GAL1 did not significantly differ between COVID-19 severity suggesting that a portion of the increased sialylation on IgM is due to the α−2,3 sialyltransferase ST3GAL4 (**Supplemental Table 4**). When IgM N-glycans were digested with the exoglycosidase Neuraminidase S, Specifically cleaving α−2,3-linked sialic acids, we detect a significant reduction in the A3G3S3 glycan species and a concomitant increase in the A3G3S2 abundance (**Supplemental Fig. 5**). Because ST6GALNAC2 adds an α−2,3 linked sialic acid to the O-glycans expressed on leukocyte cell surfaces, it is unlikely to add sialic acid to IgM [68]. However, the increased ST6GALNAC2 expression in the severe COVID-19 cohort PBMCs may reflect a reduced propensity for leukocytes to migrate into tissues due to sialic acid blocking P-/L-selectin ligand affinity [69]. Lastly, we report that a summation of all the sialic acids (S1, S2, and S3) from IgM positively correlated with the expression of ST3GAL4 (Fig. 3B). This finding suggests a potential role for ST3GAL4 adding sialic acid to IgM, but future studies will need to confirm this phenomenon Specifically in plasma blast transcriptomic studies. All in all, the PBMC transcriptomic data aligned with our observations of IgM glycosylation alterations within the severe COVID-19 cohort.

### Clinical markers of disease severity correlate with IgM glycosylation

**Figure 4:**
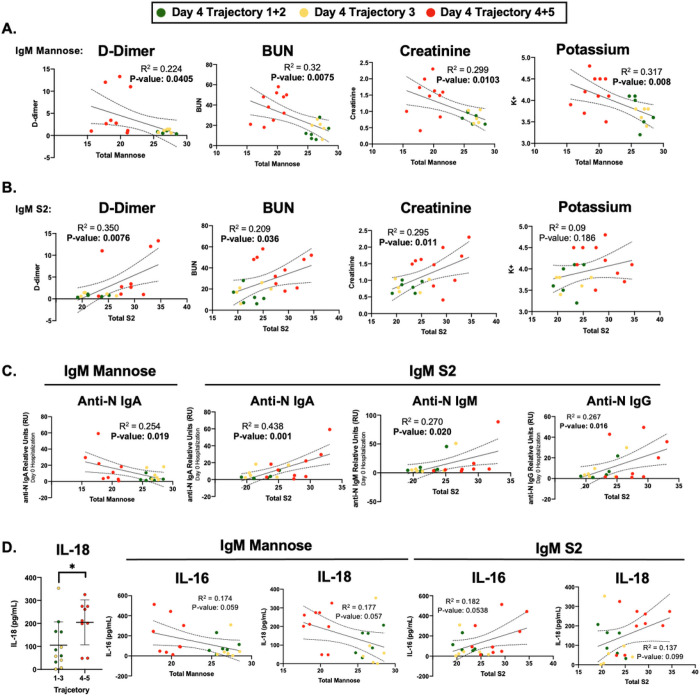
Changes in IgM N-glycosylation Associate with Clinical Markers of COVID-19 Severity **A)** Total mannose content (summation of M4-M10 and hybrid N-glycans) was correlated to hospital laboratory measurements of D-dimer, Blood urea nitrogen (BUN), creatinine, and potassium measured on day 4 of hospitalization using linear regression analysis. **B)** Total di-sialylated (S2) N-glycans were correlated with hospital laboratory measurements of D-dimer, BUN, creatinine, and potassium using simple linear regression. **C)** Anti-nucleocapsid protein (anti-N) IgA, IgM, and IgG detected from patient plasma donated at the time of hospital admission (Day 0) were correlated to IgM mannose content and S2 content. **D)** Total IL-18 measured from plasma collected on day 4 of hospitalization was compared between hospitalized trajectories 1–3 and trajectories 4 and 5. IgM mannose and S2 content were correlated with levels of the cytokines IL-16 and IL-18 as detected by Luminex 32-plex assay plasma collected on day 4 of hospitalization. Green dots identify day 4 Trajectory 1+2, yellow dots identify day 4 trajectory 3, and red dots identify day 4 trajectory 4+5 hospitalized COVID-19 cohorts. R^2^ and p-values are reported below each comparison, with bolded p-values considered statistically significant *p < 0.05 using student’s T-test.

Next, we sought to determine if the changes in IgM N-glycosylation were associated with clinical laboratory data and additional cytokine panels collected by Drexel’s IMPACC study [10, 11]. After omitting clinical parameters with less than 90% complete datasets [70], the remaining data were analyzed for correlations to IgM total mannose and S2 content using a linear regression model (**Supplemental Tables 2 and 3**). The reduction of IgM mannose in severe COVID-19 patients negatively correlated with increased D-dimer, blood urea nitrogen (BUN), creatinine, and potassium (K+) (Fig. 4A). In addition, the increased IgM S2 content positively correlated with the same clinical measurements - except for a nonsignificant correlation with potassium, p = 0.186 (Fig. 4B).

The severity of COVID-19 has also been associated with higher anti-SARS-CoV-2 IgG and IgA antibody abundance at the time of hospital admission [71]. Therefore, we sought to correlate IgM mannose and S2 glycosylation with the relative abundance of anti-SARS-CoV-2 nucleocapsid (anti-N) IgA, IgM, and IgG. Anti-N IgA relative abundance negatively correlated with IgM mannose content, while the increase in IgM S2 content positively correlated with anti-N titers of IgA, IgM, and IgG relative abundance (Fig. 4C).

Lastly, we examined Luminex data from a 32-plex cytokine panel to determine if circulating cytokines were associated with the glycosylation changes observed on IgM. Interestingly, cytokines previously demonstrated to alter glycosyltransferase activity such as IFN-γ, TNF-α, IL-6, IL-17A, or IL-10 [72, 73] did not significantly correlate with either IgM mannose or S2 content (**Supplemental Tables 2 and 3**). Moreover, only the cytokine IL-18 was significantly higher between the nonsevere and severe hospitalized COVID-19 cohorts (Fig. 4D). While not statistically significant, IL-18 and IL-16 correlated positively with IgM S2 (p = 0.099 and p = 0.0538 respectively). IgM mannose content correlated negatively with IL-18 and IL-16 (p = 0.057 and p = 0.059 respectively). Taken together, the IgM glycosylation profile closely correlates with COVID-19 severity on clinical and serological parameters and the cytokines IL-16 and IL-18 may play a role in controlling downstream glycosyltransferase expression in plasma blasts during COVID-19.

### Antibody-dependent complement deposition is increased in severe COVID-19 patients

**Figure 5:**
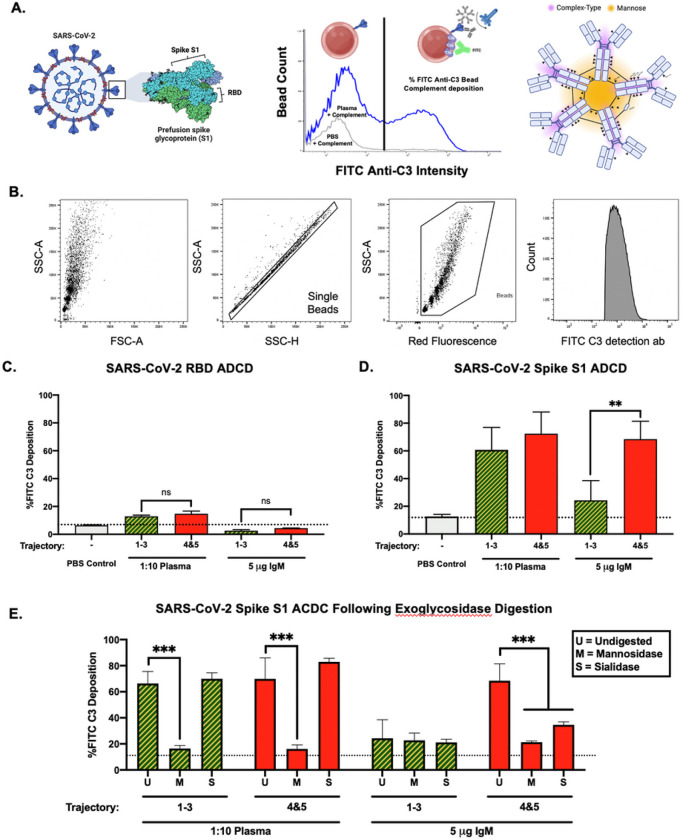
Antigen-specific complement deposition (ADCD) induced by plasma and IgM from severe and nonsevere COVID-19 cohorts **A)** Spike S1 and RBD antigen location on SARS-CoV-2 Spike glycoprotein (left), an example of how ADCD assay was quantitated using flow cytometry of plasma compared to PBS-blank sample (center), and the glycosylation of IgM pentamer displaying the c-terminus of IgM containing mannose in orange color while the purple portions of the heavy chain on IgM are complex-type N-glycans (right). **B)** Gating strategy for detection of complement deposition on fluorescent beads using flow cytometry. **C)** ADCD assay using the antigens RBD assayed in duplicate with pooled day 4 trajectory 1–3 and day 4 trajectory 4&5 plasma or IgM (left). **D)** Spike S1 antigen was assayed for ADCD with pooled day 4 trajectory 1–3 and day 4 trajectory 4&5 plasma assayed in triplicate over two experiments and the same cohorts of IgM were assayed in duplicate and then triplicate during a second experiment. **E)** Total plasma and IgM samples were digested with mannosidase (M) and sialidase (S) before ADCD analysis run in duplicate. Dotted horizontal lines refer to background binding by FITC anti-C3 antibody in PBS-only samples. Statistical significance was analyzed using one-way ANOVA, **p <0.01, ***p <0.001.

After examining clinical factors and cytokines associated with IgM N-glycosylation changes, we sought to interrogate the differences in complement deposition rates initiated by SARS-CoV-2 circulating plasma antibodies in general, and IgM Specifically. We adapted an antibody-dependent complement deposition (ADCD) assay employing fluorescent beads conjugated to a biotinylated antigen to compare complement deposition rates with SARS-CoV-2 antigens: receptor binding domain (RBD), and Spike S1 (Fig. 5A) [48]. After incubating either diluted plasma or purified IgM with antigen-coated beads, deposition of guinea pig complement was detected using flow cytometry (Fig. 5B). RBD induced low ADCD in diluted nonsevere and severe plasma, aligning with previously reported ADCD trends [74, 75] (Fig. 5C). Further, purified IgM did not induce complement deposition above the PBS background control (dotted line). However, the spike S1 antigen induced significantly higher ADCD in both plasma and purified IgM assay cohorts (Fig. 5D). Plasma from severe patients deposited higher levels of complement compared to nonsevere COVID-19 plasma, but not to a significant degree. However, IgM from the severe COVID-19 cohort induced significantly higher levels of complement deposition compared to the nonsevere cohort IgM. Next, plasma and IgM samples were digested with a mannosidase (M) or sialidase (S) and assayed for ADCD (Fig. 5E). Mannosidase treatment significantly reduced the deposition of complement on Spike S1 antigen in both plasma and IgM samples. However, the treatment with a sialidase only reduced the deposition of complement in the severe cohort IgM. Taken together, we report that severe COVID-19 cohort IgM induces higher levels of antigen-specific complement – which could be related to the alteration in the glycosylation of its mannose or sialic acid content.

## 4. Discussion

IgG N-glycosylation and effector function have been well characterized during acute COVID-19 infection [17–19][24–28]. However, IgM antibodies also play vital roles during immune responses, promote affinity maturation, maintain hemostasis at mucosal sites including the gut and lung, and induce significantly higher levels of complement deposition compared to IgG [76]. We suggest that IgM has been overlooked as a key player during the acute COVID-19 immune response. Within the IMPACC cohort enrolled at Drexel University, we find host IgM N-glycosylation correlates with disease severity.

### IgM N-glycosylation

We report a significant decrease in total IgM mannose in patients with severe COVID-19 (trajectory 4 and 5) compared to those with nonsevere COVID-19 (trajectories 1–3). By examining the mannose and hybrid structures contributing to this decrease, we conclude IgM contains fewer (M4-M6) mannose structures during severe COVID-19. Instead, IgM in severe COVID-19 contains larger mannose structures. These conclusions are supported by decreased mannosidase MAN2A1 and MAN1A2 expression within patient PBMC mRNA glycosyltransferase (GT) expression datasets. Previously, MAN1A2 genetic variability was identified as a potential correlate with susceptibility to COVID-19 infection [77]. During severe influenza, MAN1A2 was also downregulated and predicted to be highly regulated by miRNA [78, 79]. More work into the regulation of mannosidase expression is required to confirm if the changes observed in PBMC mRNA are maintained within plasma blast cell population mRNA expression, or if SARS-CoV-2 infection of PBMCs is the main factor inducing these changes in GT expression. Furthermore, the majority of IgM mannosylation is site-specific, populating the C-terminus of IgM on the Asn-563 and Asn-402 amino acids [80]. These two glycosylation sites are positioned to potentially interact with the C1q component, and impact complement activation rates in mice and humans [81, 82]. When IgM binds to an antigen target, it converts from a “planar” to a hexagonal “dome” or “staple” configuration [83]. Based on *in situ* cryo-structures, the antigen-bound IgM confirmation binds to complement C1q close to where the IgM C-terminus mannose structures are present. More work is required to determine if human IgM mannosylation impacts the affinity of C1q binding due to steric hindrance or by interacting with the mannose-binding lectin or H-ficolin [84].

Because no studies of human IgM N-glycosylation during human viral infections have been completed, we sought to compare reports of IgM N-glycan profiles characterized during other human disease states. In ovarian cancer patients, the IgM N-glycans M7 and M8 decreased on glycosite N439 (Asn563) with concomitant increases of mono- and di-sialylated N-glycans occupying N209 (Asn332) [43]. However, the group determined that the IgG and IgA N-glycan profiles predicted patients with ovarian cancer with higher accuracy than IgM N-glycans. In contrast, we observe significant differences in IgM N-glycosylation stratifying COVID-19 disease severity with decreases only in the processed M5 and M6 N-glycans. Another group reported higher levels of sialic acid detected in IgM protein fraction isolated from cancer patient sera compared to non-cancer patients [45] while another study reported no significant glycomic response associated with either IgG or IgM N-glycan profiles following tumor ablation therapy [44]. Our findings expand upon the previous reports of a general increase in sialic acid content on IgM. These sialylated IgM N-glycans likely populate the Asn-395, Asn-332, and Asn-171 glycosylation sites and could play roles in immunomodulatory signaling. When we examined the PMBC sialyltransferase mRNA expression data, we did not observe significant changes in the ST6GAL1 mRNA levels. However, we did detect increased ST3GAL4 mRNA expression, which positively correlated with the summation of all sialic acid content on IgM.

A previous high-throughput glycomic analysis of COVID-19 patients identified increased α−2,6 and α−2,3 sialylation in the total plasma, lung, and liver tissues [85]. The group associated this change with the increase in α−2,6 sialylation of the complement proteins and heightened rates of complement deposition during severe COVID-19. However, α−2,3 sialylation also has been demonstrated to modulate immune responses. Increased ST3GAL4 expression responding to the NF-κB pathway resulted in sialylated CD44 expression, exacerbating osteoarthritis in a mouse model [86]. Further, human primary chondrocytes treated with IL-1β or TNF-α increased ST3GAL4 expression and increased cellular α−2,3 sialic acid content which resulted in cartilage homeostasis disruption [87]. ST3GAL4 activity has been associated with adding the α−2,3 sialic acid required for recognition by the Siglec-3, −8, and − 9 [88] and ST3GAL4 expression regulates the synthesis of E-, P- and L-selectin ligands vital for neutrophil adhesion by increasing the binding avidity of the surface antigen sialyl-LewisX [89]. Taken together, the increase in ST3GAL4 during COVID-19 may be exerting proinflammatory downstream effects during disease pathogenesis.

While the receptor FcμR for IgM was demonstrated to bind IgM in a glycan-independent manner [90], IgM has been separately demonstrated to impair T cell proliferation in a sialic acid-dependent manner [91]. For example, the inhibitory sialic acid-binding Ig-type lectin G (Siglec G or CD22) expressed on B-cells and the human Galetcin-9 receptor expressed on the surface of APCs have been reported to bind sialylated IgM [92, 93]. Therefore, multiple receptors on immune cells may interact with IgM with increased sialic acid content, resulting in functional consequences of the humoral immune response. More work is required in this area to better understand how the changes in IgM N-glycosylation are associated with immune signaling, effector cell function, and the adaptive immune response during severe disease.

### Comparing N-glycans from IgM to N-glycans from IgG

Both IgG and IgM are glycosylated by a set of highly regulated glycosyltransferases and glycosidases (GTs) within the Golgi of plasma blast cells. GT expression is regulated by multiple cytokine and chemokine factors during an immune response, and the regulatory factors are not fully elucidated [94]. During COVID-19, we observed significant increases in the agalactosylated N-glycans on IgG with concomitant decreases of G2 and S1. In contrast, the IgM N-glycan profile lost G0 and G1 content, instead gaining S2 and S3 sialic acid as well as acquiring larger, unprocessed mannose content. The differences in glycosylation observed between IgG and IgM from the same COVID-19 patients suggest that ST3GAL4 could add sialic acid to IgM. Because IgG contains nearly all α−2,6 sialic acid [95] and ST6GAL1 transcripts remain unchanged, the upregulation of ST3GAL4 could explain how only IgM gains sialic acid while IgG does not. This is supported by our observation that α−2,3 linked sialic acids are released from IgM A3G3S3 isomers when enzymatically digested with neuraminidase S.

### IgM N-glycan correlation with markers of severity and cytokines

Predicting COVID-19 severity continues to be important to appropriately distribute healthcare resources. Markers of severe COVID-19 infection include elevated D-dimer, blood urea nitrogen (BUN), creatinine, and circulating potassium. These markers of severity significantly correlated with the IgM N-glycosylation: mannose and S2 content. It is likely that the potassium, BUN, and creatinine reflect acute kidney injury often observed in severe COVID-19 patients [96]. In previous COVID-19 studies, mild hyperkalemia is associated with COVID-19 severity and acute kidney injury due to severe COVID-19 [97] and altered potassium levels are associated with a poorer prognosis for COVID-19 survival [98]. BUN levels obtained upon emergency room evaluation significantly correlated with COVID-19 disease severity [99]. D-dimer indicates recent coagulation cascade activation by providing a marker of clot fibrinolysis, thus indirectly reflecting circulatory thrombosis [100]. During COVID-19, D-dimer has commonly been found to be elevated in severe COVID-19 patients [4]. In addition, increased titers of IgA and IgG have been associated with a more severe COVID-19 [101]. IgM N-glycan’s significant correlation with anti-nucleocapsid (N) antibodies suggests that the mechanism of severity in part correlates with IgM N-glycosylation alterations. Taken together, the significant correlations between IgM N-glycosylation profile and markers of severity suggest a potential role or response to the pathogenesis of severe COVID-19.

Of the measured cytokines in the Luminex assays performed at Drexel’s IMPACC site, only IL-16 and IL-18 were correlated with the changes in total mannose and S2 content of IgM. Most cytokines elevated in COVID-19 are proinflammatory [102] and IL-16 and IL-18 are no exception. IL-16 has previously been associated with promoting asthma severity by increasing the release of other proinflammatory cytokines [103] and promoting T cell activation by acting as a T-cell chemoattractant after being released by monocytes [104]. No studies of COVID-19 have reported IL-16 elevation associated with the severity of the disease. However, IL-18 is known to be elevated in patients with acute respiratory distress syndrome (ARDS) resulting from influenza virus infections [105]. One study correlated the level of IL-18 determined from a genome-wide association study (GWAS) was protective against severe COVID-19, and the authors suggested that IL-18 could participate in producing IFN-γ [106]. We postulate that the glycosylation of IgM could be a downstream response to the increased levels of IL-18 and IL-16 because glycosyltransferase expression can respond to cellular stimuli such as cytokine signaling [72].

### Antigen-specific complement deposition

Overactivation of complement has been associated with mortality and morbidity from COVID-19 in severe cases [74, 107–110]. Because IgM is highly effective at inducing complement, and the N-glycans on IgM were significantly altered in severe vs nonsevere COVID-19 patients, we sought to determine if we could confirm the previously reported increases of SARS-CoV-2 antigen-specific complement deposition. The RBD antigen complement deposition was low, likely due to the lower levels of anti-RBD antibodies present during the first 10 days of COVID-19 naïve patients lacking previous vaccinations. Thus, we assayed ADCD with the spike S1 antigen and observed higher complement deposition in the severe COVID-19 cohort. Next, we sought to determine if purified IgM could activate complement for these SARS-CoV-2 antigens. Purified IgM has been previously measured for complement deposition using Guinea pig complement [111, 112], but not using COVID-19 antigens. Similar to total plasma, low levels of complement deposition were detected with purified IgM from COVID-19 patients in the RBD antigen ADCD assay. However, the Spike S1 antigen complexed with IgM from the severe cohort induced significantly higher levels of complement deposited compared to IgM from the nonsevere COVID-19 cohort. IgM interacts with complement C1q via conformational shift on the IgM antigen binding face [83, 113], thus we hypothesize that the mannose or sialic acid N-glycans could impact the rate of complement deposition. We digested plasma and IgM with a mannosidase or a sialidase and determined that the ADCD for the Spike S1 antigen was significantly reduced when IgM from severe COVID-19 was treated with either a mannosidase or a sialidase. It is intriguing to see that the severe patient IgM glycosylation could be in part responsible for promoting complement deposition during COVID-19 pathogenesis. We hypothesize that complement deposition by IgM could, in turn, promote acute respiratory distress syndrome (ARDS) or acute kidney injury (AKI) observed in severe COVID-19 patients.

## 5. Limitations

This report analyzed relatively small cohorts from Drexel’s IMPACC study. Larger studies are required to confirm these findings. Furthermore, this cohort was collected early in 2020 when COVID-19 was predominantly driven by the Wuhan strain. Patients at this time lacked access to life-saving vaccines, antiviral medications, and rapid testing. Therefore, newer variants of the virus, more effective treatments, and vaccination may alter the characteristics of severe COVID-19 patient IgM N-glycosylation. One extraneous source of N-glycans is the IgM pentamer J-chain, however, only one out of the ~ 60 N-glycans per IgM pentamer is associated with the J-chain and thus this potential N-glycan contribution was ignored during data analysis. We have also not determined the ratio of hexamers and pentamers of IgM, however, this ratio could confound the reported increased complement activity. We also analyzed total IgM, limited by our detection method requiring at least 4μg of IgM to obtain adequate fluorescent and mass spectrometry signal-to-noise ratios for N-glycan identification. Antigen-specific glycopeptide mapping of IgM N-glycans using an LC-MS/MS platform would provide more accurate information about the specific immune response to severe COVID-19 infections.

## 6. Conclusion

IgM N-glycosylation changes in interesting and unexpected ways compared to IgG N-glycans in severe COVID-19 patients. The identification, quantification, and correlation of the IgM N-glycan profile within a well-characterized cohort provided opportunities to learn more about how the human immune system responds to acute viral infections. We align glycosyltransferase expression to the increased mannose complexity and sialic acid content on IgM and contrast these findings to what is canonically observed in IgG N-glycan profiles from patients with severe COVID-19. We correlate the IgM N-glycan profile to markers of disease severity and report that spike S1 specific complement deposition driven by IgM may contribute to severe COVID-19 pathophysiology. A better understanding of IgM N-glycosylation could one day result in novel therapeutics to reduce the severity of acute infectious diseases in humans. Taken together, this data opens the field for immunoglobulin M to be characterized during other infectious disease states.

## IMPACC Steering Committee:

- Clinical & Data Coordinating Center (CDCC); Precision Vaccines Program, Boston Children’s Hospital: Al Ozonoff, PhD, Joann Diray-Arce, PhD.
- Benaroya Research Institute: Matthew C. Altman, MD.
- The University of Texas at Austin: Lauren I. R. Ehrlich, PhD, Esther Melamed, MD, PhD.
- Icahn School of Medicine at Mount Sinai: Ana Fernandez Sesma, PhD, Viviana Simon, MD, PhD.
- Stanford University: Bali Pulendran, PhD, Kari C. Nadeau, MD, PhD, Mark M Davis, PhD.
- Case Western Reserve University and University Hospitals of Cleveland: Grace A. McComsey, MD, Rafick Sekaly, PhD.
- Drexel University/Tower Health Hospital: Charles B. Cairns, MD, Elias K. Haddad, PhD.
- Boston Clinical Site: Precision Vaccines Program, Boston Children’s Hospital, Brigham and Women’s Hospital, and Harvard Medical School: Lindsey R. Baden, MD, Ofer Levy, MD, PhD.
- David Geffen School of Medicine at the University of California Los Angeles: Joanna Schaenman, MD, PhD, Elaine F. Reed, PhD.
- Yale School of Medicine, and Yale School of Public Health: Albert C. Shaw, MD, PhD, David A. Hafler, MD, Ruth R. Montgomery, PhD, Steven H. Kleinstein, PhD.
- Emory University: Nadine Rouphael, MD.
- MyOwnMed, Inc. Bethesda, MD
- National Institute of Allergy and Infectious Diseases/National Institutes of Health: Patrice M. Becker, MD, Alison D. Augustine, PhD.
- University of California San Francisco School of Medicine: Carolyn S. Calfee, MD, David J. Erle, MD.
- Baylor College of Medicine, and the Center for Translational Research on Inflammatory Diseases, Michael E. DeBakey VA Medical Center: David B. Corry, MD, Farrah Kheradmand, MD.
- University of Florida/University of South Florida: Mark A. Atkinson, PhD, Scott C. Brakenridge, MD.
- Oklahoma University Health Sciences Center: Nelson I Agudelo Higuita, MD, Jordan P. Metcalf, MD.
- Oregon Health & Science University: Catherine L. Hough, MD, William B. Messer, MD, PhD.
- University of Arizona: Monica Kraft, MD, Chris Bime, MD.
- La Jolla Institute for Immunology: Bjoern Peters, PhD.
- Centre for Heart Lung Innovation, Providence Research, St. Paul’s Hospital, and the PROOF Centre of Excellence.

Clinical & Data Coordinating Center (CDCC) (Precision Vaccines Program, Boston Children’s Hospital)

Al Ozonoff, PhD, Carly E. Milliren, MPH, Joann Diray-Arce, PhD, Caitlin Syphurs, MPH, Kerry McEnaney, BS, Brenda Barton, BSN, RN, Claudia Lentucci, PhD, Mehmet Saluvan, PhD, Ana C. Chang, MS, Annmarie Hoch, BA, Marisa Albert, BSN, RN, Tanzia Shaheen, MS, MPH, Alvin T. Kho, PhD, Shanshan Liu, MS, MPH, Sanya Thomas, MBBS, Jing Chen, PhD, Maimouna D. Murphy, Mitchell Cooney, BA, Arash Nemati Hayati, PhD, Robert Bryant, BA, James Abraham, MS.

## IMPACC Data Analysis Group:

- Clinical & Data Coordinating Center (CDCC); Precision Vaccines Program, Boston Children’s Hospital: Al Ozonoff, PhD, Joann Diray-Arce, PhD.
- Benaroya Research Institute: Naresh Doni Jayavelu, PhD, Matthew C. Altman, MD, Scott Presnell, PhD, Tomasz Jancsyk, MS.
- The University of Texas at Austin: Cole Maguire, BS.
- Icahn School of Medicine at Mount Sinai: Jingjing Qi, MS, Brian Lee, BS.
- Stanford University.
- Case Western Reserve University and University Hospitals of Cleveland: Slim Fourati, PhD.
- Drexel University/Tower Health Hospital: Charles B. Cairns, MD.
- Precision Vaccines Program, Boston Children’s Hospital, Brigham and Women’s Hospital, and Harvard Medical School.
- David Geffen School of Medicine at the University of California Los Angeles.
- Yale School of Medicine, and Yale School of Public Health: Denise A. Esserman, PhD, Leying Guan, PhD, Steven H. Kleinstein, PhD, Jeremy Gygi, BS, Shrikant Pawar, PhD, Anderson Brito, PhD
- Emory University.
- MyOwnMed, Inc. Bethesda, MD
- National Institute of Allergy and Infectious Diseases/National Institutes of Health.
- University of California San Francisco School of Medicine: Gabriela K. Fragiadakis, PhD, Ravi Patel, PhD.
- Baylor College of Medicine, and the Center for Translational Research on Inflammatory Diseases, Michael E. DeBakey VA Medical Center.
- University of Florida/University of South Florida.
- Oklahoma University Health Sciences Center.
- Oregon Health & Science University.
- University of Arizona.
- La Jolla Institute for Immunology: Bjoern Peters, PhD, James A. Overton, PhD, Randi Vita, MD, Kerstin Westendorf, PhD.
- Centre for Heart Lung Innovation, Providence Research, St. Paul’s Hospital, and the PROOF Centre of Excellence: Casey P. Shannon, BSc.

## IMPACC Core Laboratory:

- Clinical & Data Coordinating Center (CDCC); Precision Vaccines Program, Boston Children’s Hospital: Joann Diray-Arce, PhD.
- Benaroya Research Institute: Matthew C. Altman, MD, Bernard Khor, MD, PhD.
- The University of Texas at Austin.
- Icahn School of Medicine at Mount Sinai: Florian Krammer, PhD, Harm van Bakel, PhD, Adeeb Rahman, PhD, Daniel Stadlbauer, PhD, Jayeeta Dutta, Hui Xie, MS, Seunghee Kim-Schulze, PhD, Ana Silvia Gonzalez-Reiche, PhD, Adriana van de Guchte, MS, Juan Manuel Carreño, PhD, Gagandeep Singh, PhD, Ariel Raskin, BA, Johnstone Tcheou, BS, Dominika Bielak, BA, Hisaaki Kawabata, BA, Brian Lee, BS, Geoffrey Kelly, MS, Manishkumar Patel,MS, Hui Xie, MS, Kai Nie, MS, Temima Yellin, BA, Miriam Fried, BA, Leeba Sullivan, BA, Sara Morris, BA.
- Stanford University: Holden T. Maecker, PhD.
- Case Western Reserve University and University Hospitals of Cleveland: Scott Sieg, PhD.
- Drexel University/Tower Health Hospital.
- Precision Vaccines Program, Boston Children’s Hospital, Brigham and Women’s Hospital, and Harvard Medical School: Hanno Steen, PhD, Patrick van Zalm, PhD, Benoit Fatou, PhD, Kevin Mendez, PhD, Jessica Lasky-Su, DSc, MS, Scott R. Hutton, PhD, Greg Michelotti, PhD, Kari Wong, PhD, Meenakshi Jha, MSc, Arthur Viode, PhD.
- David Geffen School of Medicine at the University of California Los Angeles.
- Yale School of Medicine, and Yale School of Public Health: Albert C. Shaw, MD, PhD, Yujiao Zhao, PhD, Charles Dela Cruz, MD, PhD, Ruth R. Montgomery, PhD.
- Emory University: Steven E. Bosinger, PhD, Arun K. Boddapati, MS, Greg K. Tharp, MS, Kathryn L. Pellegrini, PhD, Elizabeth Beagle, BS, David Cowan, BS, Sydney Hamilton, BS, Susan Pereira Ribeiro, PhD, Thomas Hodder, BS.
- MyOwnMed, Inc. Bethesda, MD.
- National Institute of Allergy and Infectious Diseases/National Institutes of Health. Lindsey B. Rosen, PhD, Serena Lee, BS.
- University of California San Francisco School of Medicine: Charles R. Langelier, MD, PhD, Michael R. Wilson, MD, Ravi Dandekar, MS, Bonny Alvarenga, BA, Jayant Rajan, MD, PhD, Walter Eckalbar, PhD, Andrew W. Schroeder, MPH, Alexandra Tsitsiklis, PhD, Eran Mick, PhD, Yanedth Sanchez Guerrero, PhD, Christina Love, BA, Lenka Maliskova, MS, Michael Adkisson, BS.
- Baylor College of Medicine, and the Center for Translational Research on Inflammatory Diseases, Michael E. DeBakey VA Medical Center.
- University of Florida/University of South Florida.
- Oklahoma University Health Sciences Center.
- Oregon Health & Science University.
- University of Arizona.
- La Jolla Institute for Immunology.
- Centre for Heart Lung Innovation, Providence Research, St. Paul’s Hospital, and the PROOF Centre of Excellence.
